# Pediatric Medical Subspecialist Use in Outpatient Settings

**DOI:** 10.1001/jamanetworkopen.2023.50379

**Published:** 2024-01-04

**Authors:** Christopher B. Forrest, Candice P. Chen, Eliana M. Perrin, Christopher J. Stille, Ruth Cooper, Katherine Harris, Qian Luo, Mitchell G. Maltenfort, Lauren E. Parlett

**Affiliations:** 1 Applied Clinical Research Center, Children’s Hospital of Philadelphia, Philadelphia, Pennsylvania; 2 Fitzhugh Mullan Institute for Health Workforce Equity, Milken Institute School of Public Health, George Washington University, Washington, DC; 3 Johns Hopkins University Schools of Medicine and Nursing, Baltimore, Maryland; 4 Deparment of Pediatrics, University of Colorado School of Medicine and Children’s Hospital Colorado, Aurora, Colorado; 5 Health and Medicine Division, National Academies of Sciences, Engineering, and Medicine, Washington, DC; 6 Carelon Research, Wilmington, Delaware

## Abstract

**Importance:**

A first step toward understanding whether pediatric medical subspecialists are meeting the needs of the nation’s children is describing rates of use and trends over time.

**Objectives:**

To quantify rates of outpatient pediatric medical subspecialty use.

**Design, Setting, and Participants:**

This repeated cross-sectional study of annual subspecialist use examined 3 complementary data sources: electronic health records from PEDSnet (8 large academic medical centers [January 1, 2010, to December 31, 2021]); administrative data from the Healthcare Integrated Research Database (HIRD) (14 commercial health plans [January 1, 2011, to December 31, 2021]); and administrative data from the Transformed Medicaid Statistical Information System (T-MSIS) (44 state Medicaid programs [January 1, 2016, to December 31, 2019]). Annual denominators included 493 628 to 858 551 patients younger than 21 years with a general pediatric visit in PEDSnet; 5 million beneficiaries younger than 21 years enrolled for at least 6 months in HIRD; and 35 million Medicaid or Children’s Health Insurance Program beneficiaries younger than 19 years enrolled for any amount of time in T-MSIS.

**Exposure:**

Calendar year and type of medical subspecialty.

**Main Outcomes and Measures:**

Annual number of children with at least 1 completed visit to any pediatric medical subspecialist in an outpatient setting per population. Use rates excluded visits in emergency department or inpatient settings.

**Results:**

Among the study population, the proportion of girls was 51.0% for PEDSnet, 51.1% for HIRD, and 49.3% for T-MSIS; the proportion of boys was 49.0% for PEDSnet, 48.9% for HIRD, and 50.7% for T-MSIS. The proportion of visits among children younger than 5 years was 37.4% for PEDSnet, 20.9% for HIRD, and 26.2% for T-MSIS; most patients were non-Hispanic Black (29.7% for PEDSnet and 26.1% for T-MSIS) or non-Hispanic White (44.9% for PEDSnet and 43.2% for T-MSIS). Annual rates for PEDSnet ranged from 18.0% to 21.3%, which were higher than rates for HIRD (range, 7.9%-10.4%) and T-MSIS (range, 7.6%-8.6%). Subspecialist use increased in the HIRD commercial health plans (annual relative increase of 2.4% [95% CI, 1.6%-3.1%]), but rates were essentially flat in the other data sources (PEDSnet, −0.2% [95% CI, −1.1% to 0.7%]; T-MSIS, −0.7% [95% CI, −6.5% to 5.5%]). The flat PEDSnet growth reflects a balance between annual use increases among those with commercial insurance (1.2% [95% CI, 0.3%-2.1%]) and decreases in use among those with Medicaid (−0.9% [95% CI, −1.6% to −0.2%]).

**Conclusions and Relevance:**

The findings of this cross-sectional study suggest that among children, 8.6% of Medicaid beneficiaries, 10.4% of those with commercial insurance, and 21.3% of those whose primary care is received in academic health systems use pediatric medical subspecialty care each year. There was a small increase in rates of subspecialty use among children with commercial but not Medicaid insurance. These data may help launch innovations in the primary-specialty care interface.

## Key Points

**Question:**

How have rates of outpatient pediatric medical subspecialist use changed overall and by payer type?

**Findings:**

In this cross-sectional study that used electronic health records and health plan claims data, annual rates of subspecialist use ranged from 8% (Medicaid beneficiaries) to 21% for primary care users within academic health systems. Annual rates significantly increased for children with commercial insurance, while rates decreased for children with Medicaid coverage.

**Meaning:**

These findings suggest that differences in the use of pediatric medical subspecialists by type of payer may reflect differences in demand, health need, or access to care.

## Teaser

This cross-sectional study characterizes rates and temporal patterns of outpatient pediatric medical subspecialty use and determines the most common types of conditions treated among children and adolescents with public and commercial insurance.

## Introduction

Pediatric medical subspecialists provide medical care to children whose health problems are uncommon or atypical, have not responded well to therapy typically delivered in primary care settings, or require technical expertise such as advanced procedures for diagnosis or treatment.^1^ Subspecialists serve an essential role in the health care system by providing cognitive and procedural consultative services to patients and primary care physicians and longer-term primary-specialty shared care for individuals with chronic conditions or conditions with high medical complexity,^2^ although effectiveness of coordination across the primary-specialty care interface is uneven.^3,4^ Most pediatric medical subspecialists complete a pediatric residency and then receive additional fellowship training in a subspecialty. Other subspecialists such as pediatric dermatologists complete a residency (eg, dermatology) and then complete a pediatric specialization (eg, pediatric dermatology) during their fellowship.

Understanding whether the number and distribution of pediatric medical subspecialists are meeting the needs of the nation’s children is challenging because of a paucity of evidence regarding their frequency of use. Available data that estimated rates of primary care physician referral and subspecialist use by children predate the year 2000.^1,5^ Perceptions of an undersupply of pediatric subspecialists stemming from long wait times for appointments for some subspecialties, long distances to access care, and insurance-related limitations on subspecialty access have been difficult to confirm due to lack of available data.^6,7^

To address this evidence gap, we conducted a study using 3 complementary data sources to quantify children’s use of pediatric medical subspecialties from 2011 to 2021. Electronic health record data from large pediatric medical centers and administrative data from Medicaid and commercial plans were evaluated. This study was performed in support of a National Academies of Sciences, Engineering, and Medicine (NASEM) Committee on the Pediatric Subspecialty Workforce and Its Impact on Child Health and Well-Being that produced a consensus study report.^8^ The recently released report—funded by a coalition of sponsors—recommends actions to ensure equitable access to high-quality pediatric subspecialty care to advance the health and health care of infants, children, and adolescents.

Both the NASEM report and the present study focused on pediatric medical subspecialists, excluding pediatric surgery, anesthesiology, psychiatry, radiology, and pathology practitioners. Our overall goal was to characterize the rates and temporal patterns of outpatient pediatric medical subspecialty use and determine the most common types of conditions managed to inform action on strengthening the pediatric subspecialty system of care. A secondary goal was to contrast rates of pediatric subspecialists and internal medicine– and adult-trained (IMA) subspecialists for Medicaid beneficiaries.

## Methods

The research reported in this repeated cross-sectional study used deidentified data and was designated as nonhuman subjects research and exempt from review and the requirement for informed consent by the Children’s Hospital of Philadelphia Institutional Review Board (IRB) for PEDSnet, the NASEM IRB for the Healthcare Integrated Research Database (HIRD), and the George Washington University IRB for the Transformed Medicaid Statistical Information System (T-MSIS). This study followed the Strengthening the Reporting of Observational Studies in Epidemiology (STROBE) reporting guideline.

### PEDSnet Dataset

Electronic health record data in PEDSnet are obtained from hospital information systems and standardized to the Observational Medical Outcomes Partnership common data model.^9^ Participating institutions included Ann & Robert H. Lurie Children’s Hospital of Chicago, Chicago, Illinois; Children’s Hospital Colorado, Aurora; Children’s Hospital of Philadelphia, Philadelphia, Pennsylvania; Cincinnati Children’s Hospital Medical Center, Cincinnati, Ohio; Nationwide Children’s Hospital, Columbus, Ohio; Nemours Children’s Health, Wilmington, Delaware, and Orlando, Florida; Seattle Children’s Hospital, Seattle, Washington; and Stanford Children’s Health, Stanford, California. The study sample consisted of patients with at least 1 outpatient visit to a general pediatrician that occurred from January 1, 2010, to December 31, 2021, for patients younger than 21 years. To reduce the number of patients with missing race and ethnicity data and to evaluate possible disparities in subspecialist use by race and ethnicity, we used the Centers for Disease Control and Prevention–recommended approach for a combined race and ethnicity variable in which ethnicity was used to define Hispanic category and race was used for non-Hispanic categories.^10^ Race and ethnicity were self-reported by parents and older youths. If the patient had public insurance (ie, Medicaid or Children’s Health Insurance Program [CHIP]) at any time during a 1-year observation period, that was recorded as their payer status; otherwise, if they had commercial insurance, that was listed as payer status, and all others were listed as unknown or self-pay.

### HIRD Dataset

The HIRD includes claims data submitted for payment for services to individuals enrolled in 14 commercial health plans that are part of Elevance Health (formerly Anthem Blue Cross and Blue Shield), as well as member enrollment and professional and facility claims. Medical claims are subject to the quality control, inspection, and validation procedures performed by the plans for payment processing. This analysis used enrollment files and fully adjudicated claims with service dates between January 1, 2011, and December 31, 2021.

### T-MSIS Dataset

The T-MSIS is a national Medicaid and CHIP dataset from states, territories, and Washington, DC. The first available year of T-MSIS is 2016, and the study used data from January 1, 2016, through December 31, 2019. Other service files were used to identify clinicians providing outpatient evaluation and management services. Annual Provider files were used to match and fill in the missing National Provider Identifier when available. The final analysis included 44 states, Washington, DC, and Puerto Rico and excluded Arkansas, California, Delaware, Indiana, Minnesota, and Pennsylvania because of inadequate data quality. The T-MSIS database has known data quality challenges, which are analyzed and reported by the Centers for Medicare & Medicaid Services.^11^ A patient was included in the study sample if they were enrolled in Medicaid at any time during a given year and were younger than 19 years at the end of that year. The younger upper limit age cut point for the T-MSIS was chosen to reflect Medicaid eligibility criteria.

### Pediatric Medical Subspecialties

The study examined use of 25 subspecialties in outpatient settings. This included 14 pediatric subspecialties for which the American Board of Pediatrics (ABP) provides board certification: adolescent medicine, pediatric cardiology, child abuse pediatrics, pediatric critical care medicine, developmental-behavioral pediatrics, pediatric emergency medicine, pediatric endocrinology, pediatric gastroenterology, pediatric hematology and oncology, pediatric infectious diseases, neonatal-perinatal medicine, pediatric nephrology, pediatric pulmonology, and pediatric rheumatology. Pediatric critical care medicine and neonatal-perinatal medicine were included in overall outpatient specialist use rates, but because these specialties provide so few outpatient services, they were excluded from specialty-specific calculations. Pediatric hospital medicine was excluded because of the study’s focus on outpatient care.

We also included 5 subspecialties for which the ABP cosponsors board certification, though primarily administered by other specialty boards: hospice and palliative medicine, medical toxicology, sleep medicine, sports medicine, and pediatric transplant hepatology. An additional 6 subspecialties that are not sponsored or cosponsored by the ABP but are commonly recognized as pediatric medical subspecialties were also included: pediatric allergy and immunology, child neurology, obesity medicine, pediatric dermatology, clinical genetics, and pediatric rehabilitation medicine. The T-MSIS analysis also examined IMA medical subspecialties that corresponded to the included pediatric subspecialties, excluding those with no counterpart (eg, adolescent medicine, developmental-behavioral pediatrics, and neonatal-perinatal medicine).

### Statistical Analysis

The primary rate calculation was the annual number of children with at least 1 completed visit to a subspecialist in an outpatient setting per population. Notably, this measure excludes patients for whom a referral to a subspecialist was made but the visit was not completed. Use rates excluded visits in emergency departments or inpatient settings.

Denominator definitions differed across the 3 data sources. The PEDSnet denominators included individuals with a visit to a general pediatrician during the index or prior year and therefore constituted a primary care use-based sample. The HIRD denominators required that individuals have at least 6 months of plan enrollment in a given year. The T-MSIS calendar year denominators required any duration of enrollment in Medicaid or CHIP.

The PEDSnet analysis included annual rates of patients seeing multiple types of subspecialists—that is, having at least 1 visit with 2 or more of the 25 subspecialty types. The PEDSnet analysis also compared children insured by Medicaid and those with commercial insurance as well as differences by self-identified race and ethnicity.

To calculate rates of change over time, the aggregate data (denominator and numerator in each year) were fit as a function of year using a quasi-Poisson regression, which is a Poisson regression including an allowance for potential extra variance (overdispersion) in the data. The regression coefficient for year is the log of the mean rate ratio between consecutive years. The rate ratio was extracted as the exponential of the coefficient, and then the percentage change per year was the rate ratio minus 1 expressed as a percentage. For example, a coefficient of 0.18 is exponentiated to get a rate ratio of 1.20, which gets recorded as a mean annual percentage change of 20%. We computed 95% CIs on the annual changes in rate, and those that did not cross zero were deemed statistically significant.

To describe the most common health conditions managed by different subspecialties, we used diagnostic data from PEDSnet, combining all study years. Diagnoses were combined into a cluster if they were for the same condition (eg, all diagnoses for primary and secondary hypertension were combined into a single hypertension cluster). For each health condition, we computed the proportion of patients seen by the subspecialty who were assigned the code(s) for the condition at least once. Analyses were performed using R, version 4.3.2 (R Project for Statistical Computing).

## Results

In the study sample, there was marked growth in the size of the outpatient subspecialty sample among PEDSnet (academic medical centers) from a low level of 493 628 in 2011 to a high level of 858 551 in 2021 (Table 1). Denominators were stable for HIRD (commercial health plans) at approximately 5 million and for T-MSIS (Medicaid or CHIP) at approximately 35 million. The PEDSnet dataset included proportionally more infants, while the T-MSIS dataset had a greater share of older youth.

**Table 1.** Characteristics of Study Samples^a^

Characteristic	Dataset		
	PEDSnet	HIRD	T-MSIS
Description	EHR data from 8 pediatric academic medical centers	Commercial health plan enrollment and claims data from 14 health plans	Medicaid enrollment and claims data from 44 states, Washington, DC, and Puerto Rico
Annual sample size			
2011	493 628	5 439 618	NA
2012	513 134	5 140 695	NA
2013	540 719	5 010 522	NA
2014	606 361	5 200 574	NA
2015	666 781	5 171 149	NA
2016	712 578	5 277 759	36 623 431
2017	740 059	5 346 699	35 879 612
2018	772 591	5 150 067	35 655 637
2019	815 555	5 137 756	34 824 449
2020	820 679	5 110 555	NA
2021	858 551	4 998 694	NA
Age in calendar year 2019, y			
<1	125 573 (15.4)	294 986 (5.7)	1 823 887 (5.2)
1-4	179 600 (22.0)	779 358 (15.2)	7 320 077 (21.0)
5-11	287 541 (35.3)	1 577 204 (30.7)	12 672 766 (36.4)
12-17	199 186 (24.4)	1 592 128 (31.0)	9 663 000 (27.7)
18-20	23 655 (2.9)	894 080 (17.4)	3 766 164 (10.8)
Sex			
Boys	800 313 (49.0)	2 514 031 (48.9)	17 660 985 (50.7)
Girls	833 983 (51.0)	2 623 725 (51.1)	17 155 063 (49.3)
Race and ethnicity^b^			
Asian or Pacific Islander	92 452 (6.4)	NA	1 018 738 (3.9)
Hispanic	221 794 (15.4)	NA	6 800 616 (26.2)
Non-Hispanic Black	427 375 (29.7)	NA	6 760 286 (26.1)
Non-Hispanic White	647 626 (44.9)	NA	11 198 938 (43.2)
Multiracial	51 959 (3.6)	NA	137 409 (0.5)
Payer in 2019^c^			
Commercial	343 507 (55.5)	5 137 756 (100)	0
Public	268 479 (43.4)	0	32 545 218 (100)
Self-pay	7016 (1.1)	0	0

Abbreviations: EHR, electronic health record; HIRD, Healthcare Integrated Research Database; NA, not applicable; T-MSIS, Transformed Medicaid Statistical Information System.

^a^ Unless otherwise indicated, data are expressed as No. (%) of patients.

^b^ A total of 193 090 patients (11.8% of total) in the PEDSnet sample and 8 908 462 (25.6% of total) in the T-MSIS sample had missing race and ethnicity data. These patients were excluded from the race and ethnicity distributions.

^c^ A total of 196 553 patients (24.1% of total) in the PEDSnet sample had unknown payer data in 2019. These patients were excluded from the payer distribution.

Using 2019 as a representative year across the 3 datasets, we found that the proportion of the samples younger than 5 years was 37.4% for PEDSnet, 20.9% for HIRD, and 26.2% for T-MSIS. The proportion of girls was 51.0% for PEDSnet, 51.1% for HIRD, and 49.3% for T-MSIS; the proportion of boys was 49.0% for PEDSnet, 48.9% for HIRD, and 50.7% for T-MSIS. Data on race and ethnicity were not available for HIRD. For PEDSnet, 6.4% were Asian or Pacific Islander; 15.4%, Hispanic; 29.7%, non-Hispanic Black; 44.9%, non-Hispanic White; and 3.6%, multiracial. For T-MSIS, 3.9% were Asian or Pacific Islander; 26.2%, Hispanic; 26.1%, non-Hispanic Black; 43.2%, non-Hispanic White; and 0.5%, multiracial. Annual rates of subspecialist use for PEDSnet ranged from 18.0% to 21.3%, which was double the rates for HIRD (range, 7.9%-10.4%) and T-MSIS (range, 7.6%-8.6%) (Figure).

**Figure.**
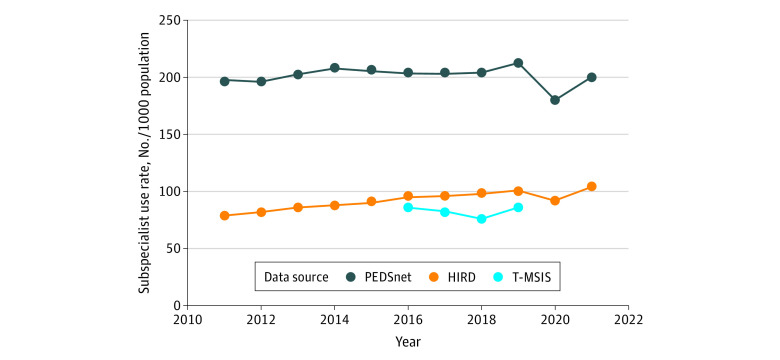
Annual Outpatient Pediatric Medical Subspecialist Use Rates, 2011 to 2021 Annual rates of pediatric medical subspecialist use in outpatient settings were computed for the PEDSnet (primary care users within academic health centers), Healthcare Integrated Research Database (HIRD; commercial health plan enrollees), and Transformed Medicaid Statistical Information System (T-MSIS; Medicaid beneficiaries) datasets. Specialist use occurred when a patient had 1 or more completed visits with any of the pediatric medical subspecialties that were studied.

Both PEDSnet and HIRD showed a pandemic-related decline in use of subspecialists in 2020 with a return to baseline in 2021. There was a trend toward increasing subspecialist use in the HIRD commercial health plans (annual relative increase of 2.4% [95% CI, 1.6%-3.1%]) but flat rates over time in the other 2 data sources (PEDSnet, −0.2% [95% CI, −1.1% to 0.7%]; T-MSIS, −0.7% [95% CI, −6.5% to 5.5%]).

The flat PEDSnet growth reflects a balance between annual increased use among patients with commercial insurance (1.2% [95% CI, 0.3%-2.1%]) and decreased use among those with Medicaid (−0.9% [95% CI, −1.6% to −0.2%]). Similarly, for subspecialist use, non-Hispanic White patients had a 1.5% increase (95% CI, 0.6%-2.4%) per year, while Asian or Pacific Islander patients had a −2.5% decline (95% CI, −0.7% to −4.3%) per year; Hispanic patients and those identifying as multiracial each had a −1.9% per-year decline (95% CIs, −1.2% to −2.7% per year for Hispanic and −1.0% to −2.7% per year for multiracial patients); and non-Hispanic Black patients had a −0.5% decline (95% CI, –0.8% to 1.9%) per year.

We computed temporal trends in subspecialist use for 13 common pediatric subspecialties (Table 2). Across all 3 data sources, pediatric rheumatology was in the top 2 subspecialties for annual rate increases. Most of the subspecialties experienced some level of increase, although the absolute levels differed by data source. To address the apparent inconsistency between flat overall rates, particularly for PEDSnet and T-MSIS, and increasing individual subspecialty rates, we used PEDSnet data to compute the annual rates of patients seeing more than 1 type of subspecialist each year. Across the study period, the rates of multiple use of subspecialists rose from 4.7% in 2011 to 6.4% in 2021.

**Table 2.** Mean Annual Change in Outpatient Subspecialist Use Rates for Selected Subspecialties by Data Source

Subspecialty	Mean annual rate of change (95% CI), %		
	PEDSnet: academic health systems, 2011-2021	HIRD: commercial plans, 2011-2021	T-MSIS: multistate Medicaid, 2016-2019
Adolescent medicine	0.6 (−0.8 to 2.0)	0.3 (−0.6 to 1.3)	−3.0 (−7.0 to 1.1)
Child neurology	0.9 (−0.6 to 2.4)	3.1 (2.5 to 3.6)	2.6 (−6.6 to 12.7)
Developmental-behavioral pediatrics	3.4 (1.7 to 5.0)	−1.8 (−3.1 to −0.4)	−0.7 (−6.7 to 5.6)
Pediatric allergy/immunology	1.4 (−0.4 to 3.2)	1.1 (0 to 2.3)	4.8 (−3.2 to 13.3)
Pediatric cardiology	0.4 (−0.6 to 1.4)	3.2 (2.3 to 4.0)	2.9 (−3.9 to 10.2)
Pediatric dermatology	4.0 (2.7 to 5.2)	3.1 (2.1 to 4.1)	3.7 (−0.7 to 8.2)
Pediatric endocrinology	1.0 (−0.3 to 2.2)	3.8 (3.2 to 4.4)	2.6 (−4.4 to 10.2)
Pediatric gastroenterology	3.0 (0.9 to 5.0)	3.6 (2.8 to 4.5)	5.1 (−3.1 to 14.1)
Pediatric hematology and oncology	0.9 (−0.2 to 1.9)	7.9 (4.2 to 11.6)	3.3 (−2.1 to 9.0)
Pediatric infectious diseases	-4.5 (−8.3 to −0.6)	8.6 (3.5 to 13.9)	0.1 (−5.2 to 5.8)
Pediatric nephrology	6.4 (0.9 to 12.3)	4.0 (3.0 to 4.9)	0.5 (−7.4 to 9.1)
Pediatric pulmonology	0 (−1.7 to 1.8)	2.5 (0.9 to 4.1)	1.6 (−9.5 to 14.0)
Pediatric rheumatology	4.1 (2.1 to 6.3)	14.4 (11.9 to 17.0)	6.3 (1.8 to 11.0)

Abbreviations: HIRD, Healthcare Integrated Research Database; T-MSIS, Transformed Medicaid Statistical Information System.

Using the T-MSIS data from 2019, we contrasted subspecialist use rates for 11 common subspecialties with both pediatric- and IMA-trained physicians (Table 3). Except for hematology and oncology, the absolute number of subspecialists with at least 1 child visit was higher for IMA- compared with pediatric-trained physicians. The IMA subspecialists more commonly provided care for adolescents rather than younger children. Rates of use of subspecialists were higher for pediatric subspecialists compared with their IMA counterparts except for allergy and immunology.

**Table 3.** Use of Pediatric vs Internal Medicine– and Adult-Trained Subspecialists in T-MSIS Dataset^a^

Subspecialty	No. of subspecialists with ≥1 pediatric visit (% total)	Subspecialist use rate per 1000 beneficiaries for calendar year 2019, age group			
		<1 y	1-11 y	12-18 y	All
Allergy and immunology					
Pediatric	242 (8.8)	0.5	1.2	0.8	1.0
Internal medicine	2522 (91.2)	2.3	11.8	10.4	10.8
Cardiology					
Pediatric	1869 (31.2)	16.1	8.0	7.9	8.4
Internal medicine	4112 (68.8)	0.4	0.4	1.0	0.6
Dermatology^b^					
Pediatric dermatology	237 (5.0)	3.4	6.5	13.4	8.7
Dermatology	4510 (95.0)	1.4	1.7	2.3	1.9
Endocrinology					
Pediatric	1072 (39.3)	3.0	5.0	9.3	6.4
Internal medicine	1659 (60.7)	0.1	0.2	0.7	0.3
Gastroenterology					
Pediatric	1261 (26.6)	8.5	7.7	7.3	7.6
Internal medicine	3471 (73.4)	0.1	0.1	0.9	0.4
Hematology and oncology					
Pediatric	1689 (53.9)	4.7	4.7	4.2	4.5
Internal medicine	1444 (46.1)	0.1	0.2	0.3	0.2
Infectious diseases					
Pediatric	641 (39.6)	4.5	2.6	1.6	2.3
Internal medicine	979 (60.4)	0.4	0.3	0.6	0.4
Nephrology					
Pediatric	516 (36.3)	2.4	2.1	2.4	2.2
Internal medicine	906 (63.7)	0.1	0.2	0.5	0.3
Neurology^b^					
Child neurology	1204 (21.9)	3.5	7.5	7.5	7.2
Neurology	4297 (78.1)	0.9	2.4	3.9	2.8
Pulmonology					
Pediatric	826 (44.9)	3.6	6.1	3.6	5.1
Internal medicine	1013 (55.1)	0.1	0.2	0.3	0.2
Rheumatology					
Pediatric	274 (22.7)	0.5	0.7	1.2	0.9
Internal medicine	934 (77.3)	<0.1	0.1	0.3	0.2

Abbreviation: T-MSIS, Transformed Medicaid Statistical Information System.

^a^ Includes Medicaid beneficiaries in calendar year 2019.

^b^ Not subspecialities of pediatrics and internal medicine, although there are within-specialty pediatric specializations (ie, pediatric dermatology and child neurology).

We identified the 3 most commonly managed health conditions for 13 pediatric subspecialties using PEDSnet data (Table 4). These 3 conditions accounted for about one-third or more patients for 8 of 13 subspecialties. Three conditions accounted for 72% of patients seen by pediatric allergists and immunologists and 50% seen by pediatric gastroenterologists. Many of the conditions across all the subspecialties were common health problems.

**Table 4.** Most Commonly Managed Health Conditions by Pediatric Medical Subspecialty in Outpatient Settings in PEDSnet Dataset^a^

Subspecialty	Commonly managed health conditions (% of patients)		
	Most common	Second most common	Third most common
Adolescent medicine	Sexually transmitted disease screening (13.2)	Obesity (10.7)	Contraception (10.2)
Child neurology	Seizures (10.6)	Developmental delay (7.1)	Migraine (6.8)
Developmental-behavioral pediatrics	Developmental delay (21.2)	Autism (15.3)	Attention deficit disorder (9.0)
Pediatric allergy and immunology	Allergic rhinitis (32.5)	Atopic dermatitis (22.2)	Food allergy (17.5)
Pediatric cardiology	Heart murmur (19.7)	Chest pain (7.5)	Palpitations (5.4)
Pediatric dermatology	Atopic dermatitis (22.6)	Inflammatory dermatosis (9.0)	Warts (7.7)
Pediatric endocrinology	Obesity (16.7)	Short stature (8.7)	Type 1 diabetes (7.1)
Pediatric gastroenterology	Constipation (19.9)	Gastroesophageal reflux (19.6)	Feeding problem (10.1)
Pediatric hematology and oncology	Anemia (11.0)	Thrombocytopenia (4.4)	Neutropenia (4.1)
Pediatric infectious diseases	Positive tuberculin test (8.9)	Inactive tuberculosis (5.1)	Fever (4.1)
Pediatric nephrology	Hypertension (23.1)	Obesity (7.0)	Proteinuria (6.6)
Pediatric pulmonology	Snoring (18.4)	Obstructive sleep apnea (15.6)	Cough (14.7)
Pediatric rheumatology	Joint pain (8.4)	Musculoskeletal pain (7.8)	Anti–nuclear antigen positive (5.0)

^a^ Diagnosis data were obtained from visits made to subspecialists from 2011 to 2021 and thus reflect the health conditions as diagnosed by the subspecialists.

## Discussion

Among children in this cross-sectional study, 8.6% of Medicaid beneficiaries, 10.4% of those commercially insured, and 21.3% of those whose primary care is received in academic health systems used pediatric medical subspecialty care each year. The rate estimates varied between data sources, which may be due to differences in access to subspecialty care by payer, geographic accessibility differences across regions of the US, type of health system, and a range of other factors that influence access and use of care. There was a small increase in rates of subspecialty use among children with commercial but not Medicaid insurance. These results do not provide any information on appropriateness of the subspecialist use, which means that it is unclear whether the temporal trend differences by payer are due to variation in demand, health needs, access, or some combination of these factors.

The PEDSnet academic medical center data demonstrate that these institutions have experienced an absolute increase in use for subspecialty care since 2011 due largely to more children receiving most of their care (primary and subspecialty) within their systems. In other words, while the subspecialist use rate per 1000 children has not increased, the number of children receiving that care has increased dramatically. Moreover, in academic medical centers, the need for multiple subspecialties for the same child has risen, pointing toward greater medical complexity among patients served. Data from inpatient settings^12^ indicate that the prevalence of complex diagnoses among children grew 45% from 2009 to 2019; most of these children also require outpatient services.

There are several reasons why the subspecialty use rates were highest in PEDSnet. First, the HIRD and T-MSIS datasets included children enrolled in health care plans, which included both users and nonusers of health care, while PEDSnet was a use-based cohort. Second, the HIRD and T-MSIS datasets included children who sought care in all types of settings, both community and academic institutions, while PEDSnet included children obtaining care from academic medical centers only. Even though we restricted the PEDSnet sample to patients who obtain primary care from those health systems, children receiving care in academic medical centers may have higher complexity and thus more intense service use. Third, PEDSnet has devoted extensive effort to validating physician subspecialty, working with each member institution to examine and improve the quality of this data element. It is possible that the other 2 datasets had lower rates because some subspecialty care may have been billed using a generic pediatric medical group code rather than a specific subspecialty group practice. The HIRD subspecialty care data relied on the clinician taxonomy documented on the claim submitted for payment. The T-MSIS analysis relied on primary specialty in the National Plan and Provider Enumeration System dataset, which may not fully capture physician subspecialties. Finally, the T-MSIS data are also limited in availability, from 2016 through 2019.

The T-MSIS analysis suggests IMA-trained subspecialists are providing health care to pediatric populations, more commonly for older children. Rates of children using IMA-trained subspecialists varied by subspecialty, suggesting different use patterns that may be related to factors such as the available supply of pediatric subspecialists. For example, the relatively small number of pediatric allergy and immunology physicians may have presented access barriers to children, who disproportionately relied on IMA-trained physicians.

### Limitations

Four limitations of this study merit discussion. First, the datasets used in these analyses are national in scope, although none was nationally representative. They were intentionally chosen to provide a set of complementary approaches for understanding patterns of pediatric subspecialist use. Second, the 3 datasets used different denominator definitions. The PEDSnet dataset was based on use of primary care physicians, while the other 2 claims datasets used months of enrollment and included both users and nonusers of primary care. These differences could have contributed to variation in rate estimation. Third, although the PEDSnet and HIRD datasets spanned the period from 2011 to 2021, the T-MSIS covered a narrower range (2016-2019). Nonetheless, the stability of all annual rates suggests that earlier T-MSIS data were likely similar to those examined in this study. Fourth, PEDSnet included patients with commercial insurance plans as well as Medicaid beneficiaries, while the other 2 datasets were restricted to one or the other financing source. The T-MSIS dataset includes a population insured by Medicaid or CHIP in over 40 states, while HIRD included beneficiaries from a single multistate health insurance company (14 health plans) and was restricted to those with commercial insurance.

## Conclusions

In this cross-sectional study, the fact that many of the common subspecialty diagnoses are health conditions regularly managed in primary care suggests the need for improved primary care–subspecialty collaborations and innovations. Referral and treatment guidelines for the most commonly referred conditions developed jointly by primary care and specialty clinicians can be used to support the busy primary care clinician, while reserving the specialist for patients most in need of specialty care. Codified guidelines can also be used to educate residents and families about the appropriateness of referral. The data provided here can serve as a launch pad for innovations in the primary-specialty care interface.

## Notes

Supplement.
Data Sharing Statement
