# Supplementary material for: Pediatric Medical Subspecialist Use in Outpatient Settings
Source: JAMA Netw Open. 2024 Jan 4;7(1):e2350379. doi: 10.1001/jamanetworkopen.2023.50379 (PMC10767594; doi:10.1001/jamanetworkopen.2023.50379)
Supplement: Supplement. — Data Sharing Statement [file jamanetwopen-e2350379-s001.pdf]

Data Sharing Statement

Forrest. Pediatric Medical Subspecialist Use in Outpatient Settings. *JAMA Netw Open*. Published January 04, 2024. doi:10.1001/jamanetworkopen.2023.50379

Data
Data available: No

Additional Information
Explanation for why data not available: The data are not from consented patients. They are from repurposed EHR and claims data. We cannot make the patient-level data available as a result. The NASEM website does contain much more detailed reports of aggregate data for all three data sources.
